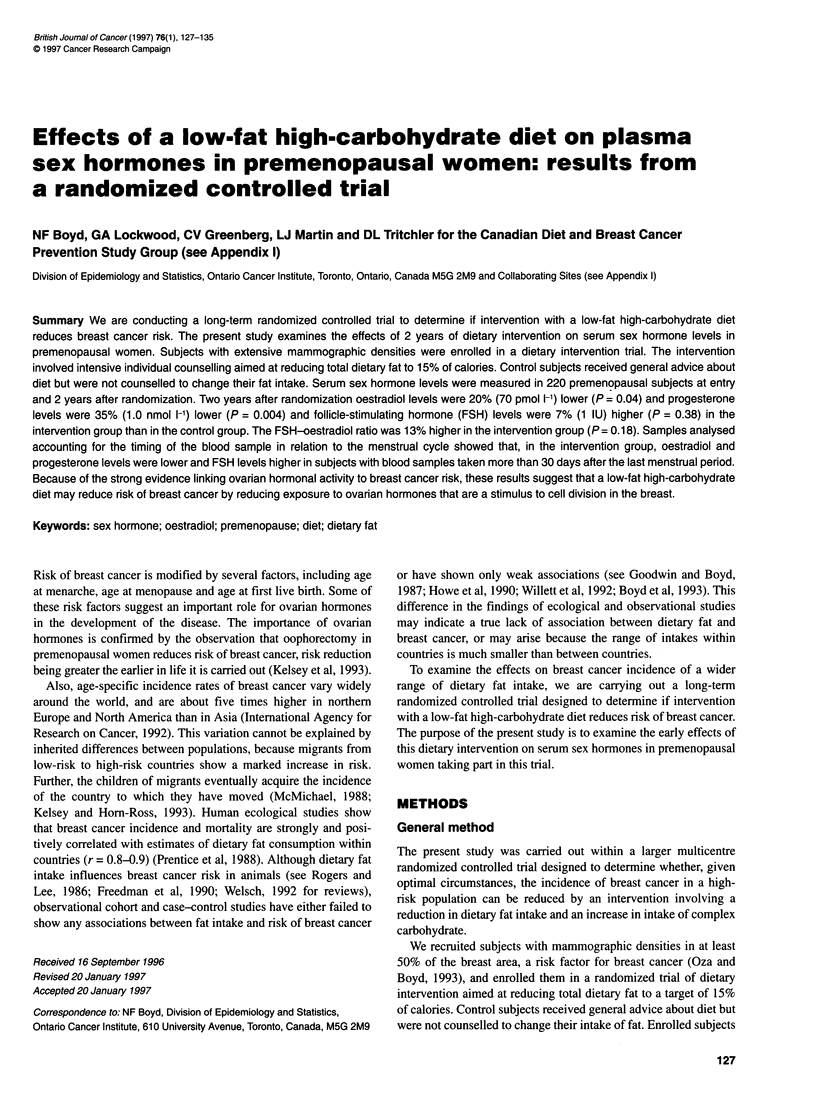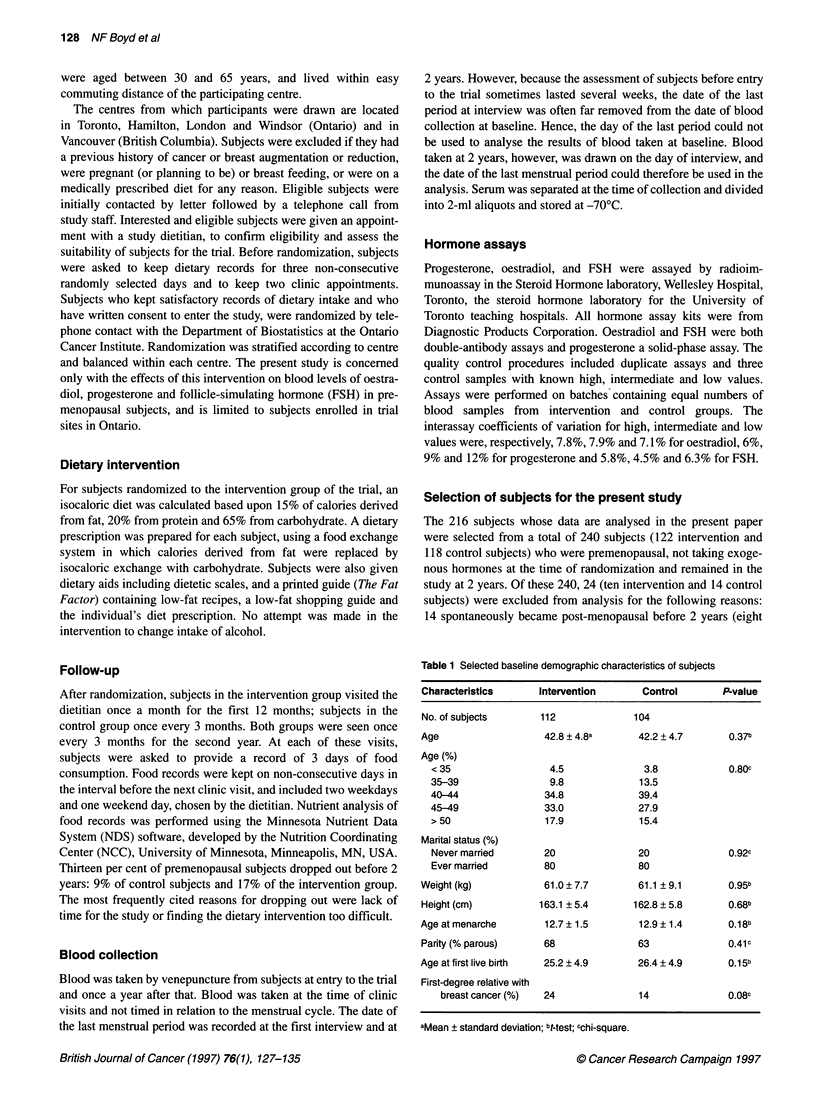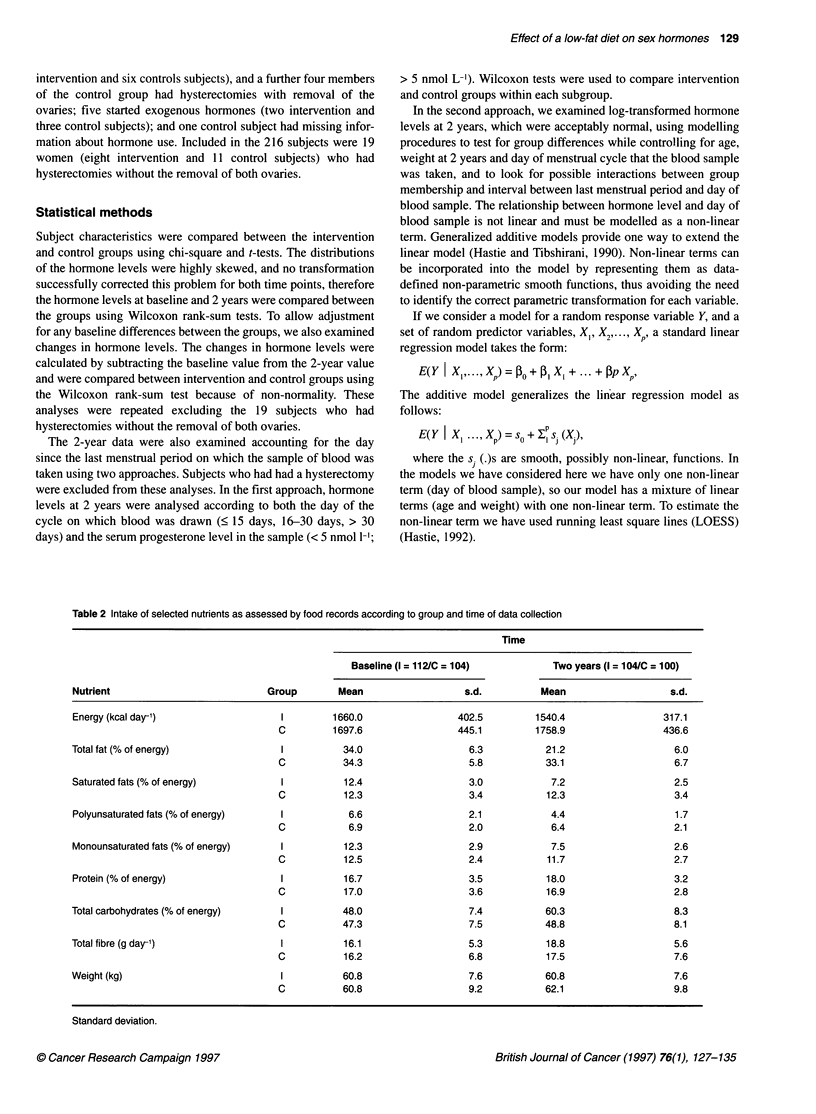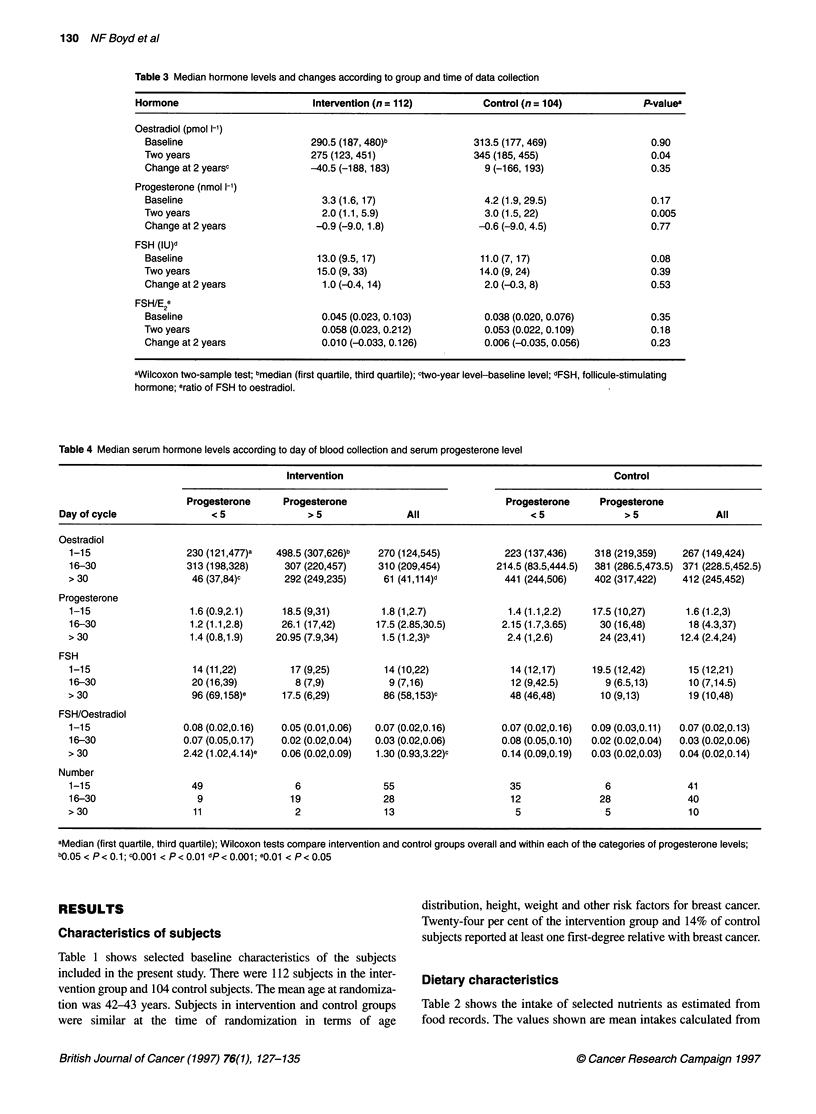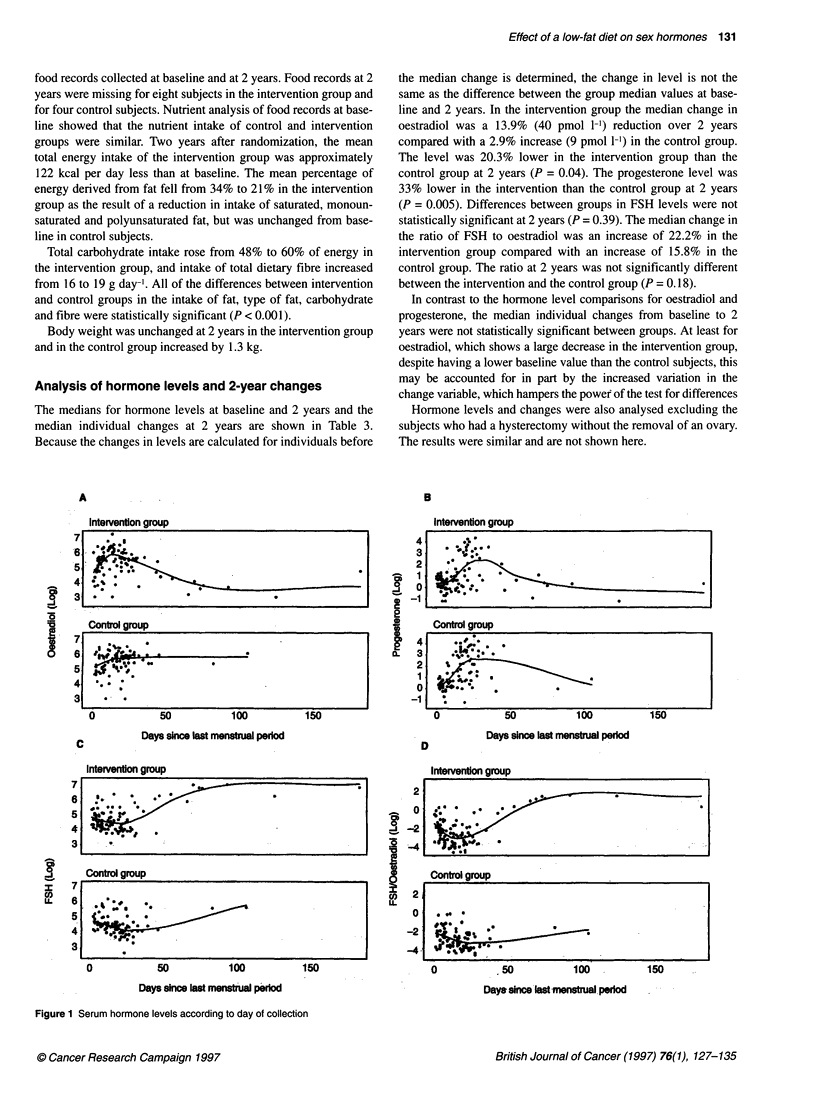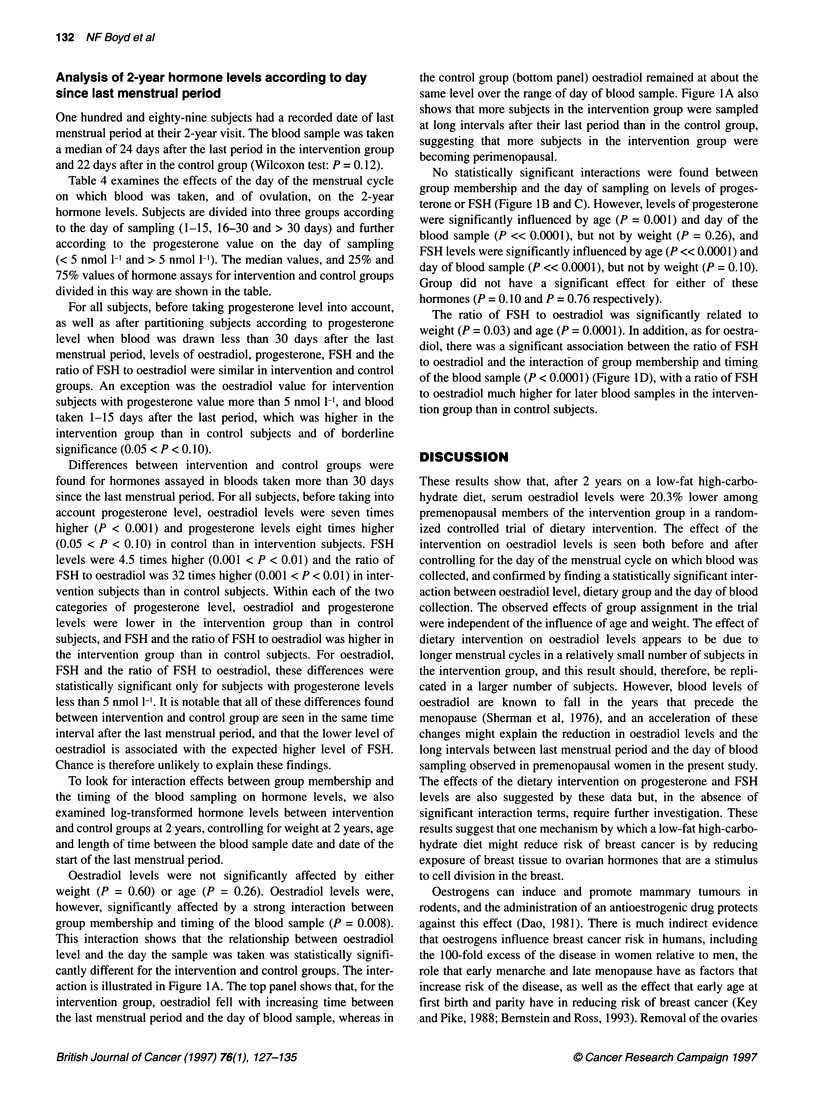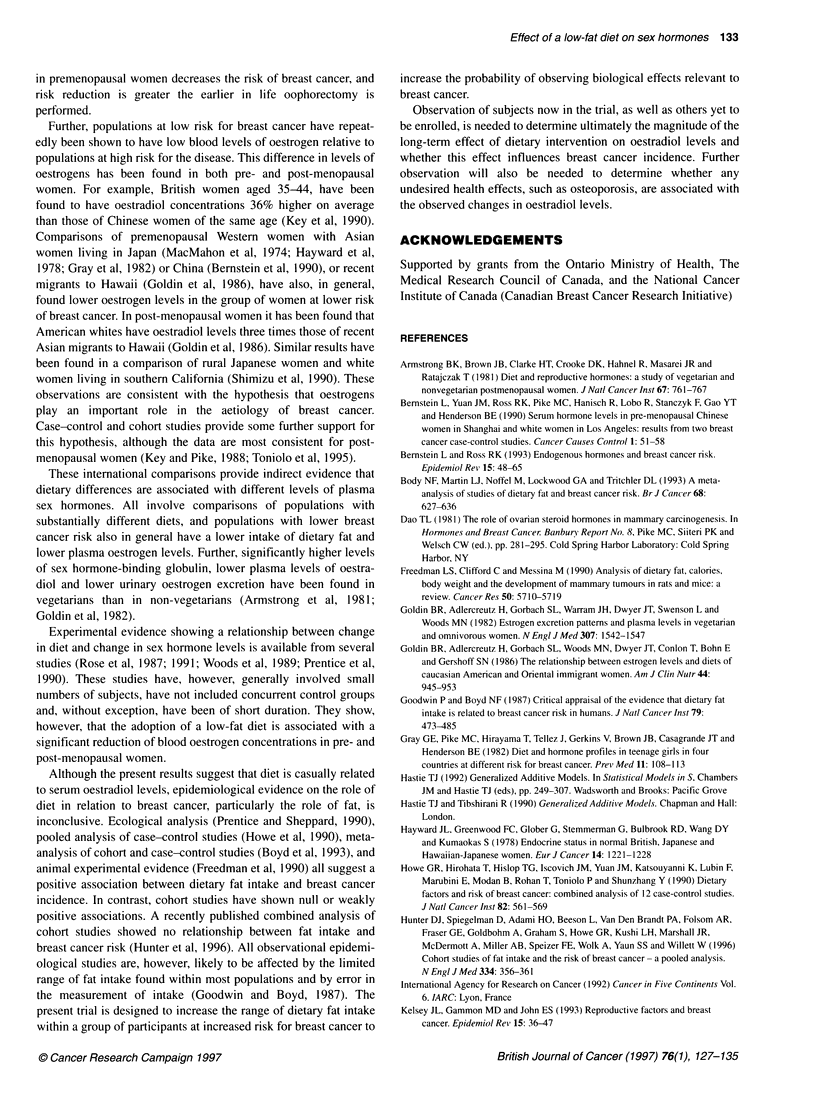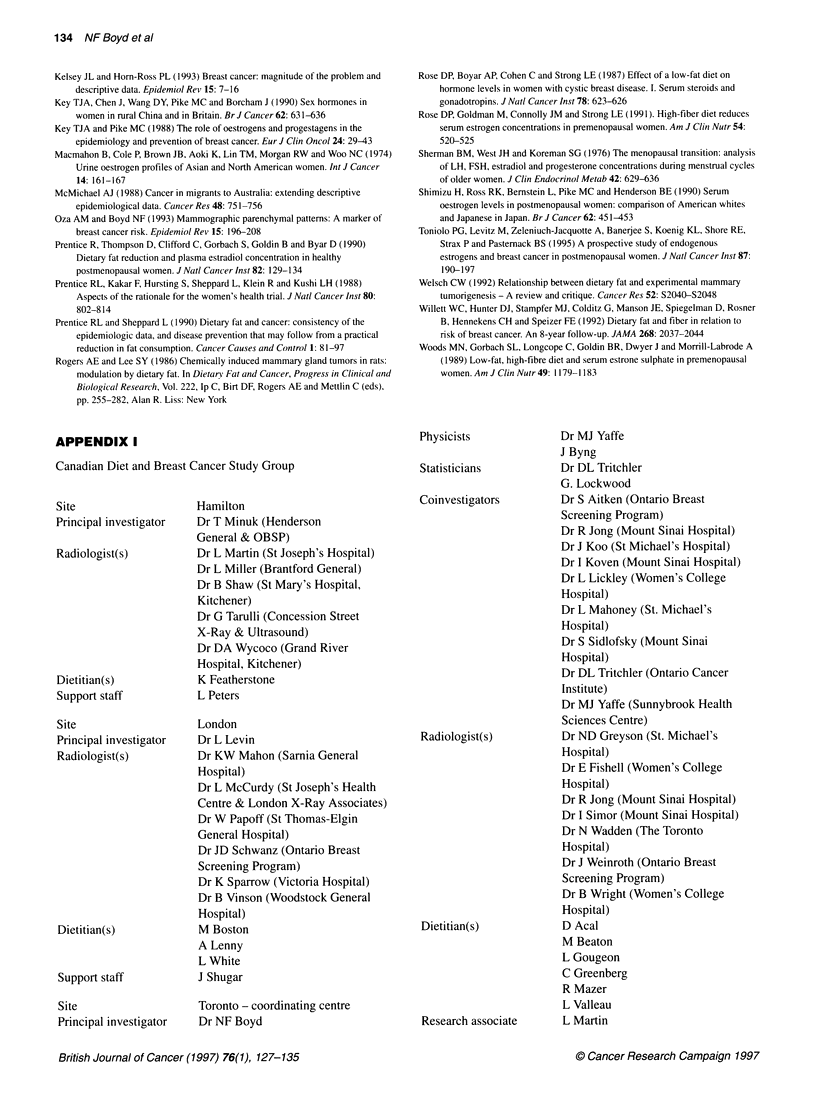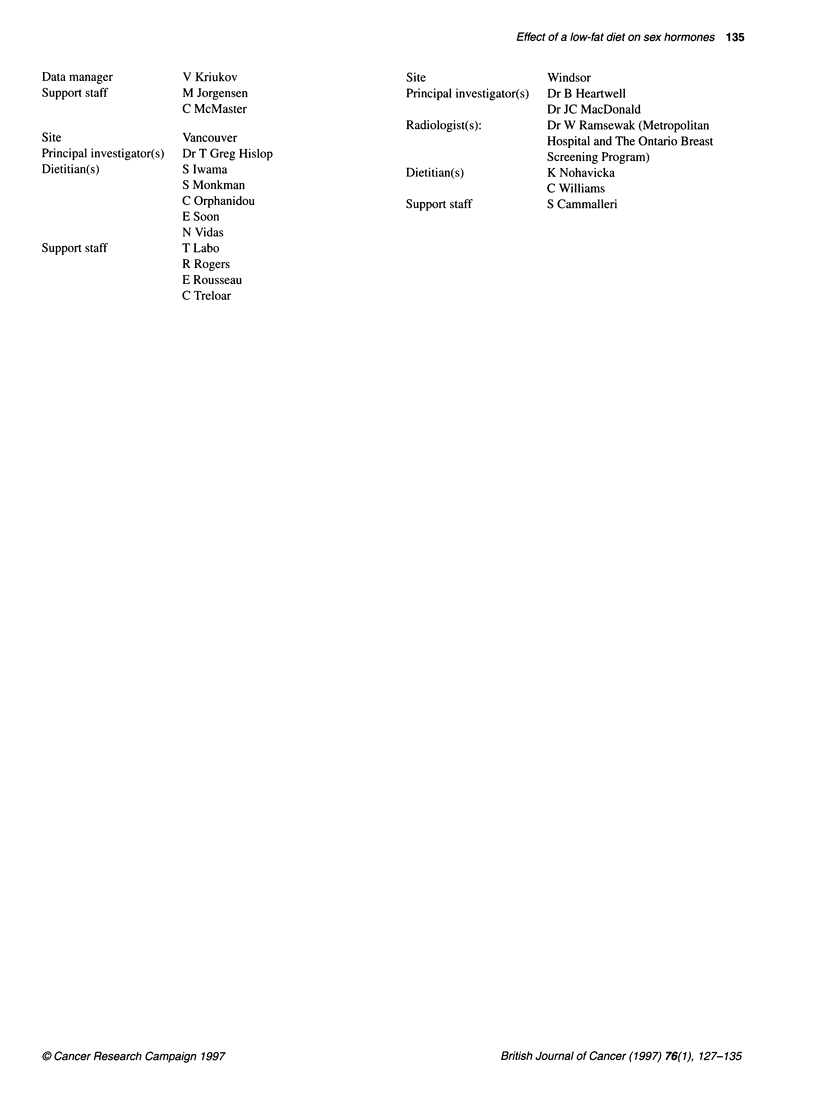# Effects of a low-fat high-carbohydrate diet on plasma sex hormones in premenopausal women: results from a randomized controlled trial. Canadian Diet and Breast Cancer Prevention Study Group.

**DOI:** 10.1038/bjc.1997.348

**Published:** 1997

**Authors:** N. F. Boyd, G. A. Lockwood, C. V. Greenberg, L. J. Martin, D. L. Tritchler

**Affiliations:** Division of Epidemiology and Statistics, Ontario Cancer Institute, Toronto, Canada.

## Abstract

We are conducting a long-term randomized controlled trial to determine if intervention with a low-fat high-carbohydrate diet reduces breast cancer risk. The present study examines the effects of 2 years of dietary intervention on serum sex hormone levels in premenopausal women. Subjects with extensive mammographic densities were enrolled in a dietary intervention trial. The intervention involved intensive individual counselling aimed at reducing total dietary fat to 15% of calories. Control subjects received general advice about diet but were not counselled to change their fat intake. Serum sex hormone levels were measured in 220 premenopausal subjects at entry and 2 years after randomization. Two years after randomization oestradiol levels were 20% (70 pmol l(-1)) lower (P = 0.04) and progesterone levels were 35% (1.0 nmol l(-1)) lower (P = 0.004) and follicle-stimulating hormone (FSH) levels were 7% (1 IU) higher (P = 0.38) in the intervention group than in the control group. The FSH-oestradiol ratio was 13% higher in the intervention group (P = 0.18). Samples analysed accounting for the timing of the blood sample in relation to the menstrual cycle showed that, in the intervention group, oestradiol and progesterone levels were lower and FSH levels higher in subjects with blood samples taken more than 30 days after the last menstrual period. Because of the strong evidence linking ovarian hormonal activity to breast cancer risk, these results suggest that a low-fat high-carbohydrate diet may reduce risk of breast cancer by reducing exposure to ovarian hormones that are a stimulus to cell division in the breast.


*British Journal of Cancer* (1997) **76**(1), 127–135
© 1997 Cancer Research Campaign

### Effects of a low-fat high-carbohydrate diet on plasma sex hormones in premenopausal women: results from a randomized controlled trial

**NF Boyd, GA Lockwood, CV Greenberg, LJ Martin and DL Tritchler for the Canadian Diet and Breast Cancer Prevention Study Group (see Appendix I)**

Division of Epidemiology and Statistics, Ontario Cancer Institute, Toronto, Ontario, Canada M5G 2M9 and Collaborating Sites (see Appendix I)

**Summary** We are conducting a long-term randomized controlled trial to determine if intervention with a low-fat high-carbohydrate diet reduces breast cancer risk. The present study examines the effects of 2 years of dietary intervention on serum sex hormone levels in premenopausal women. Subjects with extensive mammographic densities were enrolled in a dietary intervention trial. The intervention involved intensive individual counselling aimed at reducing total dietary fat to 15% of calories. Control subjects received general advice about diet but were not counselled to change their fat intake. Serum sex hormone levels were measured in 220 premenopausal subjects at entry and 2 years after randomization. Two years after randomization oestradiol levels were 20% (70 pmol $l^{-1}$) lower ($P = 0.04$) and progesterone levels were 35% (1.0 nmol $l^{-1}$) lower ($P = 0.004$) and follicle-stimulating hormone (FSH) levels were 7% (1 IU) higher ($P = 0.38$) in the intervention group than in the control group. The FSH–oestradiol ratio was 13% higher in the intervention group ($P = 0.18$). Samples analysed accounting for the timing of the blood sample in relation to the menstrual cycle showed that, in the intervention group, oestradiol and progesterone levels were lower and FSH levels higher in subjects with blood samples taken more than 30 days after the last menstrual period. Because of the strong evidence linking ovarian hormonal activity to breast cancer risk, these results suggest that a low-fat high-carbohydrate diet may reduce risk of breast cancer by reducing exposure to ovarian hormones that are a stimulus to cell division in the breast.

**Keywords:** sex hormone; oestradiol; premenopause; diet; dietary fat

Risk of breast cancer is modified by several factors, including age at menarche, age at menopause and age at first live birth. Some of these risk factors suggest an important role for ovarian hormones in the development of the disease. The importance of ovarian hormones is confirmed by the observation that oophorectomy in premenopausal women reduces risk of breast cancer, risk reduction being greater the earlier in life it is carried out (Kelsey et al, 1993).

Also, age-specific incidence rates of breast cancer vary widely around the world, and are about five times higher in northern Europe and North America than in Asia (International Agency for Research on Cancer, 1992). This variation cannot be explained by inherited differences between populations, because migrants from low-risk to high-risk countries show a marked increase in risk. Further, the children of migrants eventually acquire the incidence of the country to which they have moved (McMichael, 1988; Kelsey and Horn-Ross, 1993). Human ecological studies show that breast cancer incidence and mortality are strongly and positively correlated with estimates of dietary fat consumption within countries ($r = 0.8$–$0.9$) (Prentice et al, 1988). Although dietary fat intake influences breast cancer risk in animals (see Rogers and Lee, 1986; Freedman et al, 1990; Welsch, 1992 for reviews), observational cohort and case–control studies have either failed to show any associations between fat intake and risk of breast cancer or have shown only weak associations (see Goodwin and Boyd, 1987; Howe et al, 1990; Willett et al, 1992; Boyd et al, 1993). This difference in the findings of ecological and observational studies may indicate a true lack of association between dietary fat and breast cancer, or may arise because the range of intakes within countries is much smaller than between countries.

To examine the effects on breast cancer incidence of a wider range of dietary fat intake, we are carrying out a long-term randomized controlled trial designed to determine if intervention with a low-fat high-carbohydrate diet reduces risk of breast cancer. The purpose of the present study is to examine the early effects of this dietary intervention on serum sex hormones in premenopausal women taking part in this trial.

### METHODS

### General method

The present study was carried out within a larger multicentre randomized controlled trial designed to determine whether, given optimal circumstances, the incidence of breast cancer in a high-risk population can be reduced by an intervention involving a reduction in dietary fat intake and an increase in intake of complex carbohydrate.

We recruited subjects with mammographic densities in at least 50% of the breast area, a risk factor for breast cancer (Oza and Boyd, 1993), and enrolled them in a randomized trial of dietary intervention aimed at reducing total dietary fat to a target of 15% of calories. Control subjects received general advice about diet but were not counselled to change their intake of fat. Enrolled subjects

*Received 16 September 1996*
*Revised 20 January 1997*
*Accepted 20 January 1997*

*Correspondence to:* NF Boyd, Division of Epidemiology and Statistics, Ontario Cancer Institute, 610 University Avenue, Toronto, Canada, M5G 2M9

were aged between 30 and 65 years, and lived within easy commuting distance of the participating centre.

The centres from which participants were drawn are located in Toronto, Hamilton, London and Windsor (Ontario) and in Vancouver (British Columbia). Subjects were excluded if they had a previous history of cancer or breast augmentation or reduction, were pregnant (or planning to be) or breast feeding, or were on a medically prescribed diet for any reason. Eligible subjects were initially contacted by letter followed by a telephone call from study staff. Interested and eligible subjects were given an appointment with a study dietitian, to confirm eligibility and assess the suitability of subjects for the trial. Before randomization, subjects were asked to keep dietary records for three non-consecutive randomly selected days and to keep two clinic appointments. Subjects who kept satisfactory records of dietary intake and who have written consent to enter the study, were randomized by telephone contact with the Department of Biostatistics at the Ontario Cancer Institute. Randomization was stratified according to centre and balanced within each centre. The present study is concerned only with the effects of this intervention on blood levels of oestradiol, progesterone and follicle-simulating hormone (FSH) in premenopausal subjects, and is limited to subjects enrolled in trial sites in Ontario.

### Dietary intervention

For subjects randomized to the intervention group of the trial, an isocaloric diet was calculated based upon 15% of calories derived from fat, 20% from protein and 65% from carbohydrate. A dietary prescription was prepared for each subject, using a food exchange system in which calories derived from fat were replaced by isocaloric exchange with carbohydrate. Subjects were also given dietary aids including dietetic scales, and a printed guide (*The Fat Factor*) containing low-fat recipes, a low-fat shopping guide and the individual's diet prescription. No attempt was made in the intervention to change intake of alcohol.

### Follow-up

After randomization, subjects in the intervention group visited the dietitian once a month for the first 12 months; subjects in the control group once every 3 months. Both groups were seen once every 3 months for the second year. At each of these visits, subjects were asked to provide a record of 3 days of food consumption. Food records were kept on non-consecutive days in the interval before the next clinic visit, and included two weekdays and one weekend day, chosen by the dietitian. Nutrient analysis of food records was performed using the Minnesota Nutrient Data System (NDS) software, developed by the Nutrition Coordinating Center (NCC), University of Minnesota, Minneapolis, MN, USA. Thirteen per cent of premenopausal subjects dropped out before 2 years: 9% of control subjects and 17% of the intervention group. The most frequently cited reasons for dropping out were lack of time for the study or finding the dietary intervention too difficult.

### Blood collection

Blood was taken by venepuncture from subjects at entry to the trial and once a year after that. Blood was taken at the time of clinic visits and not timed in relation to the menstrual cycle. The date of the last menstrual period was recorded at the first interview and at 2 years. However, because the assessment of subjects before entry to the trial sometimes lasted several weeks, the date of the last period at interview was often far removed from the date of blood collection at baseline. Hence, the day of the last period could not be used to analyse the results of blood taken at baseline. Blood taken at 2 years, however, was drawn on the day of interview, and the date of the last menstrual period could therefore be used in the analysis. Serum was separated at the time of collection and divided into 2-ml aliquots and stored at –70°C.

### Hormone assays

Progesterone, oestradiol, and FSH were assayed by radioimmunoassay in the Steroid Hormone laboratory, Wellesley Hospital, Toronto, the steroid hormone laboratory for the University of Toronto teaching hospitals. All hormone assay kits were from Diagnostic Products Corporation. Oestradiol and FSH were both double-antibody assays and progesterone a solid-phase assay. The quality control procedures included duplicate assays and three control samples with known high, intermediate and low values. Assays were performed on batches containing equal numbers of blood samples from intervention and control groups. The interassay coefficients of variation for high, intermediate and low values were, respectively, 7.8%, 7.9% and 7.1% for oestradiol, 6%, 9% and 12% for progesterone and 5.8%, 4.5% and 6.3% for FSH.

### Selection of subjects for the present study

The 216 subjects whose data are analysed in the present paper were selected from a total of 240 subjects (122 intervention and 118 control subjects) who were premenopausal, not taking exogenous hormones at the time of randomization and remained in the study at 2 years. Of these 240, 24 (ten intervention and 14 control subjects) were excluded from analysis for the following reasons: 14 spontaneously became post-menopausal before 2 years (eight

**Table 1** Selected baseline demographic characteristics of subjects

| Characteristics | Intervention | Control | *P*-value |
|---|---|---|---|
| No. of subjects | 112 | 104 | |
| Age | 42.8 ± 4.8[a] | 42.2 ± 4.7 | 0.37[b] |
| Age (%) | | | |
| < 35 | 4.5 | 3.8 | 0.80[c] |
| 35–39 | 9.8 | 13.5 | |
| 40–44 | 34.8 | 39.4 | |
| 45–49 | 33.0 | 27.9 | |
| > 50 | 17.9 | 15.4 | |
| Marital status (%) | | | |
| Never married | 20 | 20 | 0.92[c] |
| Ever married | 80 | 80 | |
| Weight (kg) | 61.0 ± 7.7 | 61.1 ± 9.1 | 0.95[b] |
| Height (cm) | 163.1 ± 5.4 | 162.8 ± 5.8 | 0.68[b] |
| Age at menarche | 12.7 ± 1.5 | 12.9 ± 1.4 | 0.18[b] |
| Parity (% parous) | 68 | 63 | 0.41[c] |
| Age at first live birth | 25.2 ± 4.9 | 26.4 ± 4.9 | 0.15[b] |
| First-degree relative with breast cancer (%) | 24 | 14 | 0.08[c] |

[a]Mean ± standard deviation; [b]*t*-test; [c]chi-square.

© Cancer Research Campaign 1997

intervention and six controls subjects), and a further four members of the control group had hysterectomies with removal of the ovaries; five started exogenous hormones (two intervention and three control subjects); and one control subject had missing information about hormone use. Included in the 216 subjects were 19 women (eight intervention and 11 control subjects) who had hysterectomies without the removal of both ovaries.

### Statistical methods

Subject characteristics were compared between the intervention and control groups using chi-square and *t*-tests. The distributions of the hormone levels were highly skewed, and no transformation successfully corrected this problem for both time points, therefore the hormone levels at baseline and 2 years were compared between the groups using Wilcoxon rank-sum tests. To allow adjustment for any baseline differences between the groups, we also examined changes in hormone levels. The changes in hormone levels were calculated by subtracting the baseline value from the 2-year value and were compared between intervention and control groups using the Wilcoxon rank-sum test because of non-normality. These analyses were repeated excluding the 19 subjects who had hysterectomies without the removal of both ovaries.

The 2-year data were also examined accounting for the day since the last menstrual period on which the sample of blood was taken using two approaches. Subjects who had had a hysterectomy were excluded from these analyses. In the first approach, hormone levels at 2 years were analysed according to both the day of the cycle on which blood was drawn (≤ 15 days, 16–30 days, > 30 days) and the serum progesterone level in the sample (< 5 nmol $l^{-1}$; > 5 nmol $L^{-1}$). Wilcoxon tests were used to compare intervention and control groups within each subgroup.

In the second approach, we examined log-transformed hormone levels at 2 years, which were acceptably normal, using modelling procedures to test for group differences while controlling for age, weight at 2 years and day of menstrual cycle that the blood sample was taken, and to look for possible interactions between group membership and interval between last menstrual period and day of blood sample. The relationship between hormone level and day of blood sample is not linear and must be modelled as a non-linear term. Generalized additive models provide one way to extend the linear model (Hastie and Tibshirani, 1990). Non-linear terms can be incorporated into the model by representing them as data-defined non-parametric smooth functions, thus avoiding the need to identify the correct parametric transformation for each variable.

If we consider a model for a random response variable $Y$, and a set of random predictor variables, $X_1, X_2, \ldots, X_p$, a standard linear regression model takes the form:

$$E(Y \mid X_1, \ldots, X_p) = \beta_0 + \beta_1 X_1 + \ldots + \beta p X_p,$$

The additive model generalizes the linear regression model as follows:

$$E(Y \mid X_1 \ldots, X_p) = s_0 + \Sigma_1^p s_j (X_j),$$

where the $s_j$ (.)s are smooth, possibly non-linear, functions. In the models we have considered here we have only one non-linear term (day of blood sample), so our model has a mixture of linear terms (age and weight) with one non-linear term. To estimate the non-linear term we have used running least square lines (LOESS) (Hastie, 1992).

**Table 2** Intake of selected nutrients as assessed by food records according to group and time of data collection

| | | Time | | | |
|---|---|---|---|---|---|
| | | Baseline (I = 112/C = 104) | | Two years (I = 104/C = 100) | |
| Nutrient | Group | Mean | s.d. | Mean | s.d. |
| Energy (kcal $day^{-1}$) | I | 1660.0 | 402.5 | 1540.4 | 317.1 |
| | C | 1697.6 | 445.1 | 1758.9 | 436.6 |
| Total fat (% of energy) | I | 34.0 | 6.3 | 21.2 | 6.0 |
| | C | 34.3 | 5.8 | 33.1 | 6.7 |
| Saturated fats (% of energy) | I | 12.4 | 3.0 | 7.2 | 2.5 |
| | C | 12.3 | 3.4 | 12.3 | 3.4 |
| Polyunsaturated fats (% of energy) | I | 6.6 | 2.1 | 4.4 | 1.7 |
| | C | 6.9 | 2.0 | 6.4 | 2.1 |
| Monounsaturated fats (% of energy) | I | 12.3 | 2.9 | 7.5 | 2.6 |
| | C | 12.5 | 2.4 | 11.7 | 2.7 |
| Protein (% of energy) | I | 16.7 | 3.5 | 18.0 | 3.2 |
| | C | 17.0 | 3.6 | 16.9 | 2.8 |
| Total carbohydrates (% of energy) | I | 48.0 | 7.4 | 60.3 | 8.3 |
| | C | 47.3 | 7.5 | 48.8 | 8.1 |
| Total fibre (g $day^{-1}$) | I | 16.1 | 5.3 | 18.8 | 5.6 |
| | C | 16.2 | 6.8 | 17.5 | 7.6 |
| Weight (kg) | I | 60.8 | 7.6 | 60.8 | 7.6 |
| | C | 60.8 | 9.2 | 62.1 | 9.8 |

Standard deviation.

© Cancer Research Campaign 1997 

**Table 3** Median hormone levels and changes according to group and time of data collection

| Hormone | Intervention ($n$ = 112) | Control ($n$ = 104) | $P$-value[a] |
|---|---|---|---|
| Oestradiol (pmol $l^{-1}$) | | | |
| Baseline | 290.5 (187, 480)[b] | 313.5 (177, 469) | 0.90 |
| Two years | 275 (123, 451) | 345 (185, 455) | 0.04 |
| Change at 2 years[c] | –40.5 (–188, 183) | 9 (–166, 193) | 0.35 |
| Progesterone (nmol $l^{-1}$) | | | |
| Baseline | 3.3 (1.6, 17) | 4.2 (1.9, 29.5) | 0.17 |
| Two years | 2.0 (1.1, 5.9) | 3.0 (1.5, 22) | 0.005 |
| Change at 2 years | –0.9 (–9.0, 1.8) | –0.6 (–9.0, 4.5) | 0.77 |
| FSH (IU)[d] | | | |
| Baseline | 13.0 (9.5, 17) | 11.0 (7, 17) | 0.08 |
| Two years | 15.0 (9, 33) | 14.0 (9, 24) | 0.39 |
| Change at 2 years | 1.0 (–0.4, 14) | 2.0 (–0.3, 8) | 0.53 |
| FSH/$E_2$[e] | | | |
| Baseline | 0.045 (0.023, 0.103) | 0.038 (0.020, 0.076) | 0.35 |
| Two years | 0.058 (0.023, 0.212) | 0.053 (0.022, 0.109) | 0.18 |
| Change at 2 years | 0.010 (–0.033, 0.126) | 0.006 (–0.035, 0.056) | 0.23 |

[a]Wilcoxon two-sample test; [b]median (first quartile, third quartile); [c]two-year level–baseline level; [d]FSH, follicule-stimulating hormone; [e]ratio of FSH to oestradiol.

**Table 4** Median serum hormone levels according to day of blood collection and serum progesterone level

| | Intervention | | | Control | | |
|---|---|---|---|---|---|---|
| Day of cycle | Progesterone < 5 | Progesterone > 5 | All | Progesterone < 5 | Progesterone > 5 | All |
| Oestradiol | | | | | | |
| 1–15 | 230 (121,477)[a] | 498.5 (307,626)[b] | 270 (124,545) | 223 (137,436) | 318 (219,359) | 267 (149,424) |
| 16–30 | 313 (198,328) | 307 (220,457) | 310 (209,454) | 214.5 (83.5,444.5) | 381 (286.5,473.5) | 371 (228.5,452.5) |
| > 30 | 46 (37,84)[c] | 292 (249,235) | 61 (41,114)[d] | 441 (244,506) | 402 (317,422) | 412 (245,452) |
| Progesterone | | | | | | |
| 1–15 | 1.6 (0.9,2.1) | 18.5 (9,31) | 1.8 (1,2.7) | 1.4 (1.1,2.2) | 17.5 (10,27) | 1.6 (1.2,3) |
| 16–30 | 1.2 (1.1,2.8) | 26.1 (17,42) | 17.5 (2.85,30.5) | 2.15 (1.7,3.65) | 30 (16,48) | 18 (4.3,37) |
| > 30 | 1.4 (0.8,1.9) | 20.95 (7.9,34) | 1.5 (1.2,3)[b] | 2.4 (1,2.6) | 24 (23,41) | 12.4 (2.4,24) |
| FSH | | | | | | |
| 1–15 | 14 (11,22) | 17 (9,25) | 14 (10,22) | 14 (12,17) | 19.5 (12,42) | 15 (12,21) |
| 16–30 | 20 (16,39) | 8 (7,9) | 9 (7,16) | 12 (9,42.5) | 9 (6.5,13) | 10 (7,14.5) |
| > 30 | 96 (69,158)[e] | 17.5 (6,29) | 86 (58,153)[c] | 48 (46,48) | 10 (9,13) | 19 (10,48) |
| FSH/Oestradiol | | | | | | |
| 1–15 | 0.08 (0.02,0.16) | 0.05 (0.01,0.06) | 0.07 (0.02,0.16) | 0.07 (0.02,0.16) | 0.09 (0.03,0.11) | 0.07 (0.02,0.13) |
| 16–30 | 0.07 (0.05,0.17) | 0.02 (0.02,0.04) | 0.03 (0.02,0.06) | 0.08 (0.05,0.10) | 0.02 (0.02,0.04) | 0.03 (0.02,0.06) |
| > 30 | 2.42 (1.02,4.14)[e] | 0.06 (0.02,0.09) | 1.30 (0.93,3.22)[c] | 0.14 (0.09,0.19) | 0.03 (0.02,0.03) | 0.04 (0.02,0.14) |
| Number | | | | | | |
| 1–15 | 49 | 6 | 55 | 35 | 6 | 41 |
| 16–30 | 9 | 19 | 28 | 12 | 28 | 40 |
| > 30 | 11 | 2 | 13 | 5 | 5 | 10 |

[a]Median (first quartile, third quartile); Wilcoxon tests compare intervention and control groups overall and within each of the categories of progesterone levels; [b]$0.05 < P < 0.1$; [c]$0.001 < P < 0.01$ [d]$P < 0.001$; [e]$0.01 < P < 0.05$

### RESULTS

### Characteristics of subjects

Table 1 shows selected baseline characteristics of the subjects included in the present study. There were 112 subjects in the intervention group and 104 control subjects. The mean age at randomization was 42–43 years. Subjects in intervention and control groups were similar at the time of randomization in terms of age distribution, height, weight and other risk factors for breast cancer. Twenty-four per cent of the intervention group and 14% of control subjects reported at least one first-degree relative with breast cancer.

### Dietary characteristics

Table 2 shows the intake of selected nutrients as estimated from food records. The values shown are mean intakes calculated from

© Cancer Research Campaign 1997

food records collected at baseline and at 2 years. Food records at 2 years were missing for eight subjects in the intervention group and for four control subjects. Nutrient analysis of food records at baseline showed that the nutrient intake of control and intervention groups were similar. Two years after randomization, the mean total energy intake of the intervention group was approximately 122 kcal per day less than at baseline. The mean percentage of energy derived from fat fell from 34% to 21% in the intervention group as the result of a reduction in intake of saturated, monounsaturated and polyunsaturated fat, but was unchanged from baseline in control subjects.

Total carbohydrate intake rose from 48% to 60% of energy in the intervention group, and intake of total dietary fibre increased from 16 to 19 g day$^{-1}$. All of the differences between intervention and control groups in the intake of fat, type of fat, carbohydrate and fibre were statistically significant ($P < 0.001$).

Body weight was unchanged at 2 years in the intervention group and in the control group increased by 1.3 kg.

### Analysis of hormone levels and 2-year changes

The medians for hormone levels at baseline and 2 years and the median individual changes at 2 years are shown in Table 3. Because the changes in levels are calculated for individuals before the median change is determined, the change in level is not the same as the difference between the group median values at baseline and 2 years. In the intervention group the median change in oestradiol was a 13.9% (40 pmol l$^{-1}$) reduction over 2 years compared with a 2.9% increase (9 pmol l$^{-1}$) in the control group. The level was 20.3% lower in the intervention group than the control group at 2 years ($P = 0.04$). The progesterone level was 33% lower in the intervention than the control group at 2 years ($P = 0.005$). Differences between groups in FSH levels were not statistically significant at 2 years ($P = 0.39$). The median change in the ratio of FSH to oestradiol was an increase of 22.2% in the intervention group compared with an increase of 15.8% in the control group. The ratio at 2 years was not significantly different between the intervention and the control group ($P = 0.18$).

In contrast to the hormone level comparisons for oestradiol and progesterone, the median individual changes from baseline to 2 years were not statistically significant between groups. At least for oestradiol, which shows a large decrease in the intervention group, despite having a lower baseline value than the control subjects, this may be accounted for in part by the increased variation in the change variable, which hampers the power of the test for differences

Hormone levels and changes were also analysed excluding the subjects who had a hysterectomy without the removal of an ovary. The results were similar and are not shown here.

**Figure 1** Serum hormone levels according to day of collection

### Analysis of 2-year hormone levels according to day since last menstrual period

One hundred and eighty-nine subjects had a recorded date of last menstrual period at their 2-year visit. The blood sample was taken a median of 24 days after the last period in the intervention group and 22 days after in the control group (Wilcoxon test: $P = 0.12$).

Table 4 examines the effects of the day of the menstrual cycle on which blood was taken, and of ovulation, on the 2-year hormone levels. Subjects are divided into three groups according to the day of sampling (1–15, 16–30 and > 30 days) and further according to the progesterone value on the day of sampling (< 5 nmol $l^{-1}$ and > 5 nmol $l^{-1}$). The median values, and 25% and 75% values of hormone assays for intervention and control groups divided in this way are shown in the table.

For all subjects, before taking progesterone level into account, as well as after partitioning subjects according to progesterone level when blood was drawn less than 30 days after the last menstrual period, levels of oestradiol, progesterone, FSH and the ratio of FSH to oestradiol were similar in intervention and control groups. An exception was the oestradiol value for intervention subjects with progesterone value more than 5 nmol $l^{-1}$, and blood taken 1–15 days after the last period, which was higher in the intervention group than in control subjects and of borderline significance ($0.05 < P < 0.10$).

Differences between intervention and control groups were found for hormones assayed in bloods taken more than 30 days since the last menstrual period. For all subjects, before taking into account progesterone level, oestradiol levels were seven times higher ($P < 0.001$) and progesterone levels eight times higher ($0.05 < P < 0.10$) in control than in intervention subjects. FSH levels were 4.5 times higher ($0.001 < P < 0.01$) and the ratio of FSH to oestradiol was 32 times higher ($0.001 < P < 0.01$) in intervention subjects than in control subjects. Within each of the two categories of progesterone level, oestradiol and progesterone levels were lower in the intervention group than in control subjects, and FSH and the ratio of FSH to oestradiol was higher in the intervention group than in control subjects. For oestradiol, FSH and the ratio of FSH to oestradiol, these differences were statistically significant only for subjects with progesterone levels less than 5 nmol $l^{-1}$. It is notable that all of these differences found between intervention and control group are seen in the same time interval after the last menstrual period, and that the lower level of oestradiol is associated with the expected higher level of FSH. Chance is therefore unlikely to explain these findings.

To look for interaction effects between group membership and the timing of the blood sampling on hormone levels, we also examined log-transformed hormone levels between intervention and control groups at 2 years, controlling for weight at 2 years, age and length of time between the blood sample date and date of the start of the last menstrual period.

Oestradiol levels were not significantly affected by either weight ($P = 0.60$) or age ($P = 0.26$). Oestradiol levels were, however, significantly affected by a strong interaction between group membership and timing of the blood sample ($P = 0.008$). This interaction shows that the relationship between oestradiol level and the day the sample was taken was statistically significantly different for the intervention and control groups. The interaction is illustrated in Figure 1A. The top panel shows that, for the intervention group, oestradiol fell with increasing time between the last menstrual period and the day of blood sample, whereas in the control group (bottom panel) oestradiol remained at about the same level over the range of day of blood sample. Figure 1A also shows that more subjects in the intervention group were sampled at long intervals after their last period than in the control group, suggesting that more subjects in the intervention group were becoming perimenopausal.

No statistically significant interactions were found between group membership and the day of sampling on levels of progesterone or FSH (Figure 1B and C). However, levels of progesterone were significantly influenced by age ($P = 0.001$) and day of the blood sample ($P << 0.0001$), but not by weight ($P = 0.26$), and FSH levels were significantly influenced by age ($P << 0.0001$) and day of blood sample ($P << 0.0001$), but not by weight ($P = 0.10$). Group did not have a significant effect for either of these hormones ($P = 0.10$ and $P = 0.76$ respectively).

The ratio of FSH to oestradiol was significantly related to weight ($P = 0.03$) and age ($P = 0.0001$). In addition, as for oestradiol, there was a significant association between the ratio of FSH to oestradiol and the interaction of group membership and timing of the blood sample ($P < 0.0001$) (Figure 1D), with a ratio of FSH to oestradiol much higher for later blood samples in the intervention group than in control subjects.

### DISCUSSION

These results show that, after 2 years on a low-fat high-carbohydrate diet, serum oestradiol levels were 20.3% lower among premenopausal members of the intervention group in a randomized controlled trial of dietary intervention. The effect of the intervention on oestradiol levels is seen both before and after controlling for the day of the menstrual cycle on which blood was collected, and confirmed by finding a statistically significant interaction between oestradiol level, dietary group and the day of blood collection. The observed effects of group assignment in the trial were independent of the influence of age and weight. The effect of dietary intervention on oestradiol levels appears to be due to longer menstrual cycles in a relatively small number of subjects in the intervention group, and this result should, therefore, be replicated in a larger number of subjects. However, blood levels of oestradiol are known to fall in the years that precede the menopause (Sherman et al, 1976), and an acceleration of these changes might explain the reduction in oestradiol levels and the long intervals between last menstrual period and the day of blood sampling observed in premenopausal women in the present study. The effects of the dietary intervention on progesterone and FSH levels are also suggested by these data but, in the absence of significant interaction terms, require further investigation. These results suggest that one mechanism by which a low-fat high-carbohydrate diet might reduce risk of breast cancer is by reducing exposure of breast tissue to ovarian hormones that are a stimulus to cell division in the breast.

Oestrogens can induce and promote mammary tumours in rodents, and the administration of an antioestrogenic drug protects against this effect (Dao, 1981). There is much indirect evidence that oestrogens influence breast cancer risk in humans, including the 100-fold excess of the disease in women relative to men, the role that early menarche and late menopause have as factors that increase risk of the disease, as well as the effect that early age at first birth and parity have in reducing risk of breast cancer (Key and Pike, 1988; Bernstein and Ross, 1993). Removal of the ovaries

© Cancer Research Campaign 1997

in premenopausal women decreases the risk of breast cancer, and risk reduction is greater the earlier in life oophorectomy is performed.

Further, populations at low risk for breast cancer have repeatedly been shown to have low blood levels of oestrogen relative to populations at high risk for the disease. This difference in levels of oestrogens has been found in both pre- and post-menopausal women. For example, British women aged 35–44, have been found to have oestradiol concentrations 36% higher on average than those of Chinese women of the same age (Key et al, 1990). Comparisons of premenopausal Western women with Asian women living in Japan (MacMahon et al, 1974; Hayward et al, 1978; Gray et al, 1982) or China (Bernstein et al, 1990), or recent migrants to Hawaii (Goldin et al, 1986), have also, in general, found lower oestrogen levels in the group of women at lower risk of breast cancer. In post-menopausal women it has been found that American whites have oestradiol levels three times those of recent Asian migrants to Hawaii (Goldin et al, 1986). Similar results have been found in a comparison of rural Japanese women and white women living in southern California (Shimizu et al, 1990). These observations are consistent with the hypothesis that oestrogens play an important role in the aetiology of breast cancer. Case–control and cohort studies provide some further support for this hypothesis, although the data are most consistent for post-menopausal women (Key and Pike, 1988; Toniolo et al, 1995).

These international comparisons provide indirect evidence that dietary differences are associated with different levels of plasma sex hormones. All involve comparisons of populations with substantially different diets, and populations with lower breast cancer risk also in general have a lower intake of dietary fat and lower plasma oestrogen levels. Further, significantly higher levels of sex hormone-binding globulin, lower plasma levels of oestradiol and lower urinary oestrogen excretion have been found in vegetarians than in non-vegetarians (Armstrong et al, 1981; Goldin et al, 1982).

Experimental evidence showing a relationship between change in diet and change in sex hormone levels is available from several studies (Rose et al, 1987; 1991; Woods et al, 1989; Prentice et al, 1990). These studies have, however, generally involved small numbers of subjects, have not included concurrent control groups and, without exception, have been of short duration. They show, however, that the adoption of a low-fat diet is associated with a significant reduction of blood oestrogen concentrations in pre- and post-menopausal women.

Although the present results suggest that diet is casually related to serum oestradiol levels, epidemiological evidence on the role of diet in relation to breast cancer, particularly the role of fat, is inconclusive. Ecological analysis (Prentice and Sheppard, 1990), pooled analysis of case–control studies (Howe et al, 1990), meta-analysis of cohort and case–control studies (Boyd et al, 1993), and animal experimental evidence (Freedman et al, 1990) all suggest a positive association between dietary fat intake and breast cancer incidence. In contrast, cohort studies have shown null or weakly positive associations. A recently published combined analysis of cohort studies showed no relationship between fat intake and breast cancer risk (Hunter et al, 1996). All observational epidemiological studies are, however, likely to be affected by the limited range of fat intake found within most populations and by error in the measurement of intake (Goodwin and Boyd, 1987). The present trial is designed to increase the range of dietary fat intake within a group of participants at increased risk for breast cancer to increase the probability of observing biological effects relevant to breast cancer.

Observation of subjects now in the trial, as well as others yet to be enrolled, is needed to determine ultimately the magnitude of the long-term effect of dietary intervention on oestradiol levels and whether this effect influences breast cancer incidence. Further observation will also be needed to determine whether any undesired health effects, such as osteoporosis, are associated with the observed changes in oestradiol levels.

### ACKNOWLEDGEMENTS

Supported by grants from the Ontario Ministry of Health, The Medical Research Council of Canada, and the National Cancer Institute of Canada (Canadian Breast Cancer Research Initiative)

### REFERENCES

Armstrong BK, Brown JB, Clarke HT, Crooke DK, Hahnel R, Masarei JR and Ratajczak T (1981) Diet and reproductive hormones: a study of vegetarian and nonvegetarian postmenopausal women. *J Natl Cancer Inst* **67**: 761–767

Bernstein L, Yuan JM, Ross RK, Pike MC, Hanisch R, Lobo R, Stanczyk F, Gao YT and Henderson BE (1990) Serum hormone levels in pre-menopausal Chinese women in Shanghai and white women in Los Angeles: results from two breast cancer case-control studies. *Cancer Causes Control* **1**: 51–58

Bernstein L and Ross RK (1993) Endogenous hormones and breast cancer risk. *Epidemiol Rev* **15**: 48–65

Body NF, Martin LJ, Noffel M, Lockwood GA and Tritchler DL (1993) A meta-analysis of studies of dietary fat and breast cancer risk. *Br J Cancer* **68**: 627–636

Dao TL (1981) The role of ovarian steroid hormones in mammary carcinogenesis. In *Hormones and Breast Cancer. Banbury Report No. 8*, Pike MC, Siiteri PK and Welsch CW (ed.), pp. 281–295. Cold Spring Harbor Laboratory: Cold Spring Harbor, NY

Freedman LS, Clifford C and Messina M (1990) Analysis of dietary fat, calories, body weight and the development of mammary tumours in rats and mice: a review. *Cancer Res* **50**: 5710–5719

Goldin BR, Adlercreutz H, Gorbach SL, Warram JH, Dwyer JT, Swenson L and Woods MN (1982) Estrogen excretion patterns and plasma levels in vegetarian and omnivorous women. *N Engl J Med* **307**: 1542–1547

Goldin BR, Adlercreutz H, Gorbach SL, Woods MN, Dwyer JT, Conlon T, Bohn E and Gershoff SN (1986) The relationship between estrogen levels and diets of caucasian American and Oriental immigrant women. *Am J Clin Nutr* **44**: 945–953

Goodwin P and Boyd NF (1987) Critical appraisal of the evidence that dietary fat intake is related to breast cancer risk in humans. *J Natl Cancer Inst* **79**: 473–485

Gray GE, Pike MC, Hirayama T, Tellez J, Gerkins V, Brown JB, Casagrande JT and Henderson BE (1982) Diet and hormone profiles in teenage girls in four countries at different risk for breast cancer. *Prev Med* **11**: 108–113

Hastie TJ (1992) Generalized Additive Models. In *Statistical Models in S*, Chambers JM and Hastie TJ (eds), pp. 249–307. Wadsworth and Brooks: Pacific Grove

Hastie TJ and Tibshirani R (1990) *Generalized Additive Models*. Chapman and Hall: London.

Hayward JL, Greenwood FC, Glober G, Stemmerman G, Bulbrook RD, Wang DY and Kumaokas S (1978) Endocrine status in normal British, Japanese and Hawaiian-Japanese women. *Eur J Cancer* **14**: 1221–1228

Howe GR, Hirohata T, Hislop TG, Iscovich JM, Yuan JM, Katsouyanni K, Lubin F, Marubini E, Modan B, Rohan T, Toniolo P and Shunzhang Y (1990) Dietary factors and risk of breast cancer: combined analysis of 12 case-control studies. *J Natl Cancer Inst* **82**: 561–569

Hunter DJ, Spiegelman D, Adami HO, Beeson L, Van Den Brandt PA, Folsom AR, Fraser GE, Goldbohm A, Graham S, Howe GR, Kushi LH, Marshall JR, McDermott A, Miller AB, Speizer FE, Wolk A, Yaun SS and Willett W (1996) Cohort studies of fat intake and the risk of breast cancer – a pooled analysis. *N Engl J Med* **334**: 356–361

International Agency for Research on Cancer (1992) *Cancer in Five Continents* Vol. 6. *IARC*: Lyon, France

Kelsey JL, Gammon MD and John ES (1993) Reproductive factors and breast cancer. *Epidemiol Rev* **15**: 36–47

© Cancer Research Campaign 1997

Kelsey JL and Horn-Ross PL (1993) Breast cancer: magnitude of the problem and descriptive data. *Epidemiol Rev* **15**: 7–16

Key TJA, Chen J, Wang DY, Pike MC and Borcham J (1990) Sex hormones in women in rural China and in Britain. *Br J Cancer* **62**: 631–636

Key TJA and Pike MC (1988) The role of oestrogens and progestagens in the epidemiology and prevention of breast cancer. *Eur J Clin Oncol* **24**: 29–43

Macmahon B, Cole P, Brown JB, Aoki K, Lin TM, Morgan RW and Woo NC (1974) Urine oestrogen profiles of Asian and North American women. *Int J Cancer* **14**: 161–167

McMichael AJ (1988) Cancer in migrants to Australia: extending descriptive epidemiological data. *Cancer Res* **48**: 751–756

Oza AM and Boyd NF (1993) Mammographic parenchymal patterns: A marker of breast cancer risk. *Epidemiol Rev* **15**: 196–208

Prentice R, Thompson D, Clifford C, Gorbach S, Goldin B and Byar D (1990) Dietary fat reduction and plasma estradiol concentration in healthy postmenopausal women. *J Natl Cancer Inst* **82**: 129–134

Prentice RL, Kakar F, Hursting S, Sheppard L, Klein R and Kushi LH (1988) Aspects of the rationale for the women's health trial. *J Natl Cancer Inst* **80**: 802–814

Prentice RL and Sheppard L (1990) Dietary fat and cancer: consistency of the epidemiologic data, and disease prevention that may follow from a practical reduction in fat consumption. *Cancer Causes and Control* **1**: 81–97

Rogers AE and Lee SY (1986) Chemically induced mammary gland tumors in rats: modulation by dietary fat. In *Dietary Fat and Cancer, Progress in Clinical and Biological Research*, Vol. 222, Ip C, Birt DF, Rogers AE and Mettlin C (eds), pp. 255–282, Alan R. Liss: New York

Rose DP, Boyar AP, Cohen C and Strong LE (1987) Effect of a low-fat diet on hormone levels in women with cystic breast disease. I. Serum steroids and gonadotropins. *J Natl Cancer Inst* **78**: 623–626

Rose DP, Goldman M, Connolly JM and Strong LE (1991). High-fiber diet reduces serum estrogen concentrations in premenopausal women. *Am J Clin Nutr* **54**: 520–525

Sherman BM, West JH and Koreman SG (1976) The menopausal transition: analysis of LH, FSH, estradiol and progesterone concentrations during menstrual cycles of older women. *J Clin Endocrinol Metab* **42**: 629–636

Shimizu H, Ross RK, Bernstein L, Pike MC and Henderson BE (1990) Serum oestrogen levels in postmenopausal women: comparison of American whites and Japanese in Japan. *Br J Cancer* **62**: 451–453

Toniolo PG, Levitz M, Zeleniuch-Jacquotte A, Banerjee S, Koenig KL, Shore RE, Strax P and Pasternack BS (1995) A prospective study of endogenous estrogens and breast cancer in postmenopausal women. *J Natl Cancer Inst* **87**: 190–197

Welsch CW (1992) Relationship between dietary fat and experimental mammary tumorigenesis – A review and critique. *Cancer Res* **52**: S2040–S2048

Willett WC, Hunter DJ, Stampfer MJ, Colditz G, Manson JE, Spiegelman D, Rosner B, Hennekens CH and Speizer FE (1992) Dietary fat and fiber in relation to risk of breast cancer. An 8-year follow-up. *JAMA* **268**: 2037–2044

Woods MN, Gorbach SL, Longcope C, Goldin BR, Dwyer J and Morrill-Labrode A (1989) Low-fat, high-fibre diet and serum estrone sulphate in premenopausal women. *Am J Clin Nutr* **49**: 1179–1183

### APPENDIX I

Canadian Diet and Breast Cancer Study Group

| | |
|---|---|
| Site | Hamilton |
| Principal investigator | Dr T Minuk (Henderson General & OBSP) |
| Radiologist(s) | Dr L Martin (St Joseph's Hospital)<br>Dr L Miller (Brantford General)<br>Dr B Shaw (St Mary's Hospital, Kitchener)<br>Dr G Tarulli (Concession Street X-Ray & Ultrasound)<br>Dr DA Wycoco (Grand River Hospital, Kitchener) |
| Dietitian(s) | K Featherstone |
| Support staff | L Peters |

| | |
|---|---|
| Site | London |
| Principal investigator | Dr L Levin |
| Radiologist(s) | Dr KW Mahon (Sarnia General Hospital)<br>Dr L McCurdy (St Joseph's Health Centre & London X-Ray Associates)<br>Dr W Papoff (St Thomas-Elgin General Hospital)<br>Dr JD Schwanz (Ontario Breast Screening Program)<br>Dr K Sparrow (Victoria Hospital)<br>Dr B Vinson (Woodstock General Hospital) |
| Dietitian(s) | M Boston<br>A Lenny<br>L White |
| Support staff | J Shugar |

| | |
|---|---|
| Site | Toronto – coordinating centre |
| Principal investigator | Dr NF Boyd |
| Physicists | Dr MJ Yaffe<br>J Byng |
| Statisticians | Dr DL Tritchler<br>G. Lockwood |
| Coinvestigators | Dr S Aitken (Ontario Breast Screening Program)<br>Dr R Jong (Mount Sinai Hospital)<br>Dr J Koo (St Michael's Hospital)<br>Dr I Koven (Mount Sinai Hospital)<br>Dr L Lickley (Women's College Hospital)<br>Dr L Mahoney (St. Michael's Hospital)<br>Dr S Sidlofsky (Mount Sinai Hospital)<br>Dr DL Tritchler (Ontario Cancer Institute)<br>Dr MJ Yaffe (Sunnybrook Health Sciences Centre) |
| Radiologist(s) | Dr ND Greyson (St. Michael's Hospital)<br>Dr E Fishell (Women's College Hospital)<br>Dr R Jong (Mount Sinai Hospital)<br>Dr I Simor (Mount Sinai Hospital)<br>Dr N Wadden (The Toronto Hospital)<br>Dr J Weinroth (Ontario Breast Screening Program)<br>Dr B Wright (Women's College Hospital) |
| Dietitian(s) | D Acal<br>M Beaton<br>L Gougeon<br>C Greenberg<br>R Mazer<br>L Valleau |
| Research associate | L Martin |

© Cancer Research Campaign 1997

| | |
|---|---|
| Data manager | V Kriukov |
| Support staff | M Jorgensen |
| | C McMaster |
| | |
| Site | Vancouver |
| Principal investigator(s) | Dr T Greg Hislop |
| Dietitian(s) | S Iwama |
| | S Monkman |
| | C Orphanidou |
| | E Soon |
| | N Vidas |
| Support staff | T Labo |
| | R Rogers |
| | E Rousseau |
| | C Treloar |

| | |
|---|---|
| Site | Windsor |
| Principal investigator(s) | Dr B Heartwell |
| | Dr JC MacDonald |
| Radiologist(s): | Dr W Ramsewak (Metropolitan Hospital and The Ontario Breast Screening Program) |
| Dietitian(s) | K Nohavicka |
| | C Williams |
| Support staff | S Cammalleri |

© Cancer Research Campaign 1997